# Distribution and Antimicrobial Resistance Patterns of Aerobic Bacterial Isolates from Clinically Ill Pet Guinea Pigs (*Cavia porcellus*) in Hong Kong

**DOI:** 10.3390/ani15142042

**Published:** 2025-07-11

**Authors:** Desiree Hung, Ibrahim Elsohaby, Fraser Hill, Andrew Ferguson, Colin T. McDermott

**Affiliations:** 1 Jockey Club College of Veterinary Medicine and Life Sciences, City University of Hong Kong, Hong Kong SAR 999077, China; desirhung2-c@my.cityu.edu.hk; 2 Department of Infectious Diseases and Public Health, Jockey Club of Veterinary Medicine and Life Sciences, City University of Hong Kong, Hong Kong SAR 999077, China; ielsohab@cityu.edu.hk; 3 Centre for Applied One Health Research and Policy Advice (OHRP), City University of Hong Kong, Hong Kong SAR 999077, China; 4 CityU Veterinary Diagnostic Laboratory Co., Ltd., City University of Hong Kong, Hong Kong SAR 999077, China; fraser.hill@cityuvdl.com.hk (F.H.); andrew.ferguson@cityuvdl.com.hk (A.F.); 5 Department of Veterinary Clinical Sciences, Jockey Club College of Veterinary Medicine and Life Sciences, City University of Hong Kong, Hong Kong SAR 999077, China

**Keywords:** antimicrobial resistance, exotic pets, guinea pigs, multidrug resistance, *E. coli*, *Staphylococcus*, *Streptococcus*

## Abstract

With the increase in keeping exotic companion mammals as pets, concerns about antimicrobial resistance (AMR) and its impact on animal and human health are growing. Guinea pigs, a popular pet in Hong Kong and globally, have limited studies regarding antimicrobial culture and sensitivity results. We reviewed bacteriologic and antimicrobial sensitivity results from clinically ill pet guinea pigs from 2019 to 2023 using data from the City University Veterinary Diagnostic Laboratory. Of the 234 clinical samples from 22 veterinary clinics in Hong Kong, 134 (57.3%) showed positive bacterial growth, of which 23 (17.2%) showed mixed bacterial growth. In total, 156 bacterial isolates were identified. Gram-positive bacteria (*n* = 104, 66.7%) were most commonly recovered, representing 25 bacterial species, most commonly *Streptococcus* spp., *Staphylococcus* spp., and *Corynebacterium* spp. The majority of positive samples were from the integument (43.6%) and urinary tract (33.8%). A total of 85.9% of all isolates were resistant to at least one antimicrobial agent, with over 40% of isolates exhibiting resistance to three or more antimicrobial agents, and 27.6% were multidrug resistant (resistant to at least one agent in three or more antimicrobial classes). High resistance rates were observed for penicillin (45.6%), gentamicin (43.7%), doxycycline (42.1%), and azithromycin (36.3%). In contrast, isolates were highly susceptible to ceftazidime (84.1%), chloramphenicol (82.6%), ciprofloxacin (72.7%), and marbofloxacin (72.2%). These findings highlight the high frequency of AMR in this population of clinically ill pet guinea pigs in Hong Kong and the need for informed and judicious antimicrobial use.

## Simple Summary

Antimicrobial resistance (AMR) is an escalating global health concern, where bacteria evolve to withstand antibiotics that once effectively treated infections. This study investigated bacterial infections and AMR in pet guinea pigs in Hong Kong. We reviewed 234 medical records of ill guinea pigs from 2019 to 2023 to identify the most common bacterial infections and assess their resistance to antibiotics. Over half of the samples showed bacterial growth, with many strains resistant to commonly used antibiotics. Alarmingly, more than a quarter were multidrug-resistant, complicating treatment options. Some antibiotics traditionally considered safe for guinea pigs may also be less effective due to rising resistance levels. These findings underscore the need for responsible antibiotic use in exotic pets and suggest that pet guinea pigs may contribute to the broader spread of resistant bacteria. This information can guide veterinarians in selecting more effective treatments and increase awareness of AMR in companion animals.

## 1. Introduction

Antimicrobial resistance (AMR) is a growing concern in veterinary medicine, and the use of antibiotics in exotic pet species may contribute to the development and spread of resistant bacteria [1]. The close contact between pets and their owners may facilitate transmission of resistant bacteria, posing a potential risk to public health [2,3]. For instance, a recent study on pet rabbits in Hong Kong revealed high levels of multidrug-resistant bacterial isolates that can be transmitted from animals to pet owners [4]. Similar studies on guinea pigs have reported varying levels of AMR among bacterial isolates, with some isolates exhibiting resistance to multiple antimicrobial agents [1,4,5,6]. However, there is limited epidemiological data on the prevalence of bacterial isolates and their AMR profiles in pet guinea pigs, particularly in the Southeast Asia region.

Bacterial infections are a major cause of morbidity and mortality in pet guinea pigs and are commonly associated with poor husbandry and hygiene practices [7]. Accurate diagnosis through culture and sensitivity testing, and treatment of these infections are crucial for improving patient outcomes and promoting antimicrobial stewardship [2]. However, selecting antimicrobials for treating bacterial infections in guinea pigs can be challenging due to their unique anatomical and physiological characteristics. Guinea pigs rely on hindgut fermentation and bacterial flora for energy production, making them susceptible to antibiotic-induced dysbiosis from an overgrowth of harmful organisms [8].

Empirical antibiotic therapy is often necessary for treating bacterial infections in guinea pigs while awaiting culture and susceptibility results. The selection of appropriate first-line antibiotics should be based on the knowledge of the most common bacterial pathogens associated with specific infection sites and their expected antimicrobial susceptibility patterns. Previous literature has suggested that antibiotics such as trimethoprim/sulfonamide, fluoroquinolones, tetracyclines, chloramphenicol, and aminoglycosides may have less impact on the enteric microbial flora in guinea pigs and could be considered for empirical therapy [8]. However, the susceptibility to these antibiotics among common bacterial isolates in clinically ill guinea pigs remains poorly characterized.

In Hong Kong, guinea pigs have become a popular choice of companion animal due to their manageable size, docile nature, and suitability for urban living [9]. Although specific data on the exact number of households owning guinea pigs are limited, pet ownership surveys suggest a growing interest in keeping exotic mammals as pets [9,10]. Despite this trend, little is known about AMR in pet guinea pigs in Hong Kong or their potential role in the transmission and persistence of AMR in humans. Thus, this research aims to improve our understanding of bacterial isolates in clinically ill pet guinea pigs in Hong Kong through (1) identifying the common bacterial isolates in clinically ill pet guinea pigs in Hong Kong, and (2) determining the antimicrobial resistance patterns of these bacterial isolates.

## 2. Materials and Methods

### 2.1. Study Design and Data Extraction

**Figure 1:**
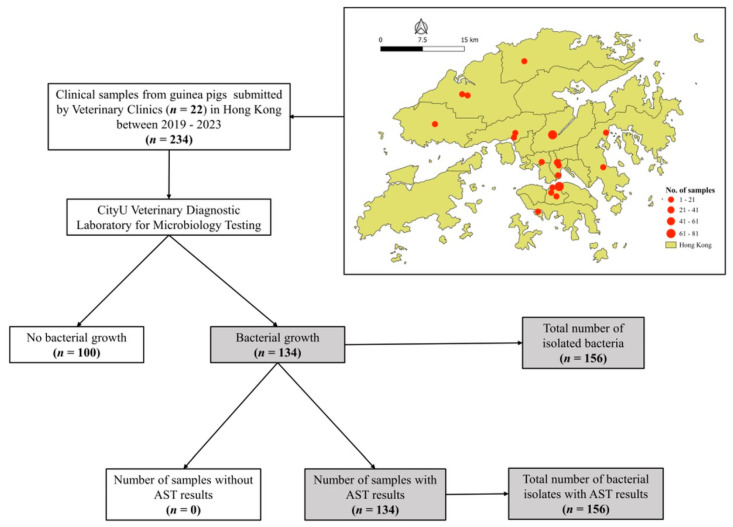
Schematic flowchart of the study design, featuring the distribution map of veterinary clinics and data extraction.

The archives of the Veterinary Diagnostic Laboratory at City University of Hong Kong (CityU VDL) were searched for electronic medical records of pet guinea pigs’ clinical samples submitted by veterinarians between January 2019 and December 2023. The submitted samples included swabs, fresh tissue, fluid samples, bladder flushes, cystocentesis, voided urine, and fecal samples collected by exotic veterinary clinics across Hong Kong (Figure 1) and were submitted specifically for microbiological and antimicrobial susceptibility testing (AST). According to the submission forms and available medical records, all guinea pigs included in this study presented with clinical illness that necessitated the submission of an aerobic culture and AST for further diagnosis. All submission forms and associated clinical data were reviewed by one author (D.H.), and the data were confirmed by a second author (C.T.M.) prior to further analysis.

In total, 234 records were downloaded, and results of aerobic bacterial culture and AST profiles (*n* = 234) were extracted. Additional information, such as the veterinary clinic’s name, animal date of birth, sex, sample type, sampling site, sampling date, and reported history of prior antibiotic administration, was also extracted from each record. However, data related to anaerobic bacterial culture, with minimum inhibitory concentration (*n* = 30) and fungal culture (*n* = 1) were excluded from the analysis.

### 2.2. Bacterial Isolation and Identification

Aerobic cultures from standard samples were inoculated onto 5% sheep blood agar (SBA), MacConkey agar (MAC), and chocolate agar (CHOC) (Thermo Fisher Scientific, Inc., Waltham, MA, USA). Urine samples were inoculated on cystine–lactose–electrolyte-deficient agar and SBA (bioMérieux, Marcy-l’Étoile, France). Tissue samples and swabs from sterile sites were inoculated into cooked meat medium as an enrichment medium, alongside SBA, MAC, and CHOC. For fecal and rectal samples, inoculation was performed on SBA, MAC, Xylosine Lysine Deoxycholate agar, and selenite broth (Thermo Fisher Scientific, Inc., Waltham, MA, USA). All samples were incubated at 37 °C for up to 48 h under aerobic conditions. Bacterial colonies were collected and identified using matrix-assisted laser desorption ionization–time-of-flight (MALDI-TOF) mass spectrometry (Bruker Daltonics, Billerica, MA, USA) and analyzed with the MALDI-TOF Biotyper software version 3.4 with the BDAL library version 1.

### 2.3. Antimicrobial Susceptibility Testing

The AST of aerobic bacterial isolates was determined using the Kirby–Bauer disc diffusion method. Antimicrobial agents tested included azithromycin, cefazolin, ceftazidime, cephalexin, chloramphenicol, ciprofloxacin, doxycycline, enrofloxacin, florfenicol, framycetin, fusidic acid, gentamicin, marbofloxacin (Liofilchem, Roseto degli Abruzzi, TE, Italy), neomycin, penicillin, tetracycline, tobramycin, and trimethoprim–sulfamethoxazole (Thermo Fisher Scientific, Inc., Waltham, MA, USA). Control strains such as *Escherichia coli* ATCC 25922, *Streptococcus pneumoniae* ATCC 49619, *Staphylococcus aureus* ATCC 25923, and *Pseudomonas aeruginosa* ATCC 27853 were used for routine quality control. Resistance rates were reported only for antimicrobials with a minimum of 30 tested isolates [11]. Results were interpreted as sensitive, intermediate, or resistant according to CLSI guidelines [11]. Intrinsic resistance to specific antimicrobials was excluded from data analysis, following the latest EUCAST expert rules on intrinsic resistance and exceptional phenotypes [12]. Only acquired resistance was analyzed and presented. The multiple antibiotic resistance (MAR) index was calculated as the ratio of the number of antimicrobials to which the isolates displayed resistance to the number of antimicrobials to which the isolates were tested [13]. Multidrug resistance (MDR) was defined as resistance to at least one agent in three or more antimicrobial classes [14].

### 2.4. Statistical Analysis

Data were analyzed using R software (v.4.2.0) for analysis and visualization, and QGIS software (v.3.30.3) was used to map the approximate locations of veterinary clinics. Categorical variables were expressed as numbers and percentages. A chi-squared test was used to assess the difference in the bacterial isolation rate. The distribution of the MAR index was assessed using the Shapiro–Wilk test, which indicated significant deviation from normality. Consequently, the non-parametric Kruskal–Wallis test was used to evaluate differences in the MAR index across variables such as sampling year, sex, age, sampling site, and bacterial species. When significant differences were detected (*p*-value  < 0.05), Dunn’s post hoc test with Bonferroni correction was applied for pairwise comparisons.

## 3. Results

### 3.1. Animals’ Demographic, Geographic, and Temporal Data

A total of 234 clinical samples from pet guinea pigs were submitted for microbiological examination and AST at CityU VDL by 22 exotic veterinary clinics in Hong Kong between January 2019 and December 2023 (Figure 1). The number of submissions varied annually, with the highest number in 2022 (33.8%), followed by 2020 (23.1%).

Integument samples (43.6%) were the most common, followed by urinary tract samples (33.8%). The majority of samples were swabs (66.7%), primarily from wounds and abscesses (Table 1). Among the guinea pigs, 64.5% were male, 34.6% female, and sex information was unavailable for 0.9%.

The guinea pigs ranged in age from 1 day to 8 years, with a mean age of 3 years. Based on age categories, 13.7% were young (<1 year), 47% were middle-aged (1–3 years), and 38.5% were older (>3 years). Recent antimicrobial use was reported in 4.7% of cases (Table 1).

### 3.2. Bacterial Isolates

Out of 234 clinical samples, 134 (57.3%) showed bacterial growth. Among these, 111 (82.8%) had single bacterial isolates, while 23 (17.2%) had mixed growth, yielding a total of 156 bacterial isolates. The bacterial isolation rate was higher in males (60.3%) than in females (36.5%), though the difference was not statistically significant (*p* = 0.16). However, the isolation rate was significantly higher in older guinea pigs compared to younger ones (*p* = 0.009).

**Figure 2:**
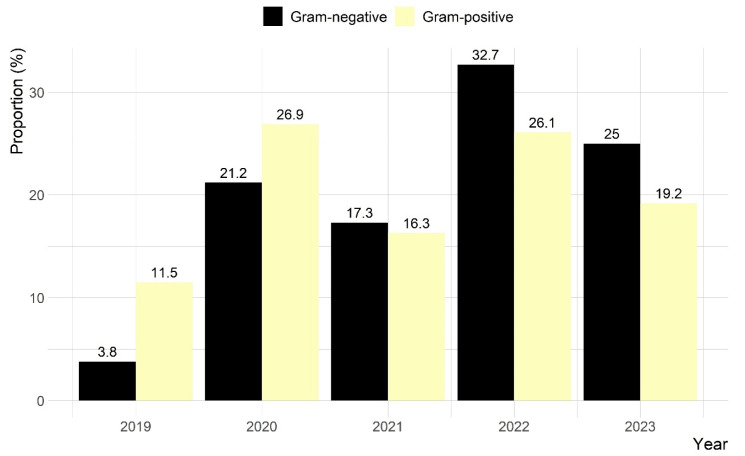
Proportions of bacterial groups from 234 guinea pig clinical samples in Hong Kong, stratified by sampling year.

**Figure 3:**
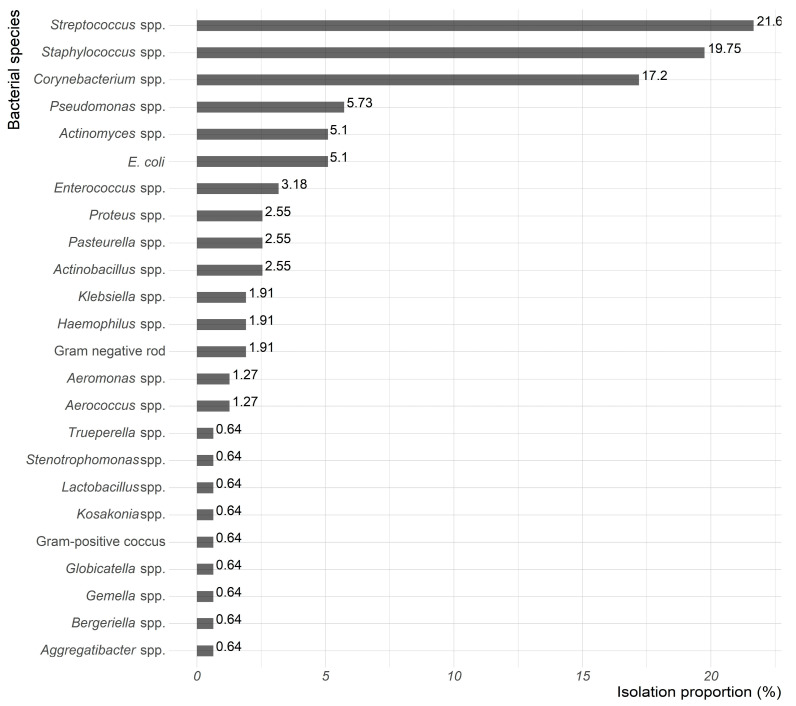
Proportions of bacterial isolates from 234 guinea pig clinical samples in Hong Kong.

Gram-positive bacteria (*n* = 104, 66.7%) were the most commonly recovered (Figure 2). The isolates comprised 25 bacterial species, with *Streptococcus* spp., *Staphylococcus* spp., and *Corynebacterium* spp. being the most frequent (Figure 3). Among these, 34 (21.6%) were *Streptococcus* spp., including 10 (29.4%) identified as *S. equi subsp zooepidemicus*. Additionally, 31 (19.8%) were *Staphylococcus* spp., with 10 (32.3%) identified as *S. aureus* and 5 (16.1%) as *S. xylosus*.

### 3.3. Antimicrobial Susceptibility

**Figure 4:**
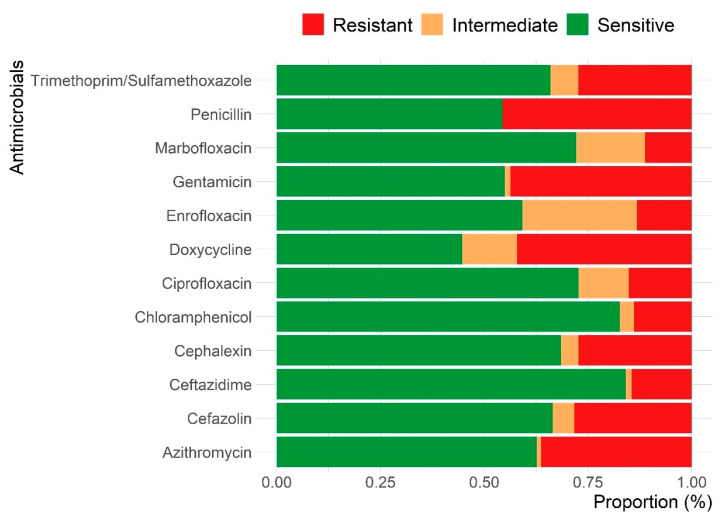
Frequency of antimicrobial resistance in bacterial isolates from 234 guinea pig clinical samples in Hong Kong. Resistance rates are reported only for antimicrobials with a minimum of 30 tested isolates, consistent with CLSI guidelines.

Antimicrobial sensitivity testing revealed that 85.9% (*n* = 134) of isolates from pet guinea pig clinical samples in Hong Kong were resistant to at least one antimicrobial agent (Figure S1). High resistance rates were observed for penicillin (45.6%), gentamicin (43.7%), doxycycline (42.1%), and azithromycin (36.3%). In contrast, isolates were highly susceptible to ceftazidime (84.1%), chloramphenicol (82.6%), ciprofloxacin (72.7%), and marbofloxacin (72.2%) (Figure 4). Among prevalent bacterial species, over 40% of isolates exhibited resistance to ≥3 antimicrobial agents, except for *Corynebacterium* spp. (Table 2). All *Enterococcus* spp. and *Pseudomonas* spp. isolates were resistant to >3 antimicrobial agents, followed by *Staphylococcus* spp. (45.2%) and *Actinomyces* spp. (42.8%). Of the 134 resistant isolates, 37 (27.6%) were MDR, with *Pseudomonas* spp. (100%), *E. coli* (62.5%), *Actinomyces* spp. (42.8%), and *Streptococcus* spp. (41.2%) showing the highest MDR levels (Table 2).

**Figure 5:**
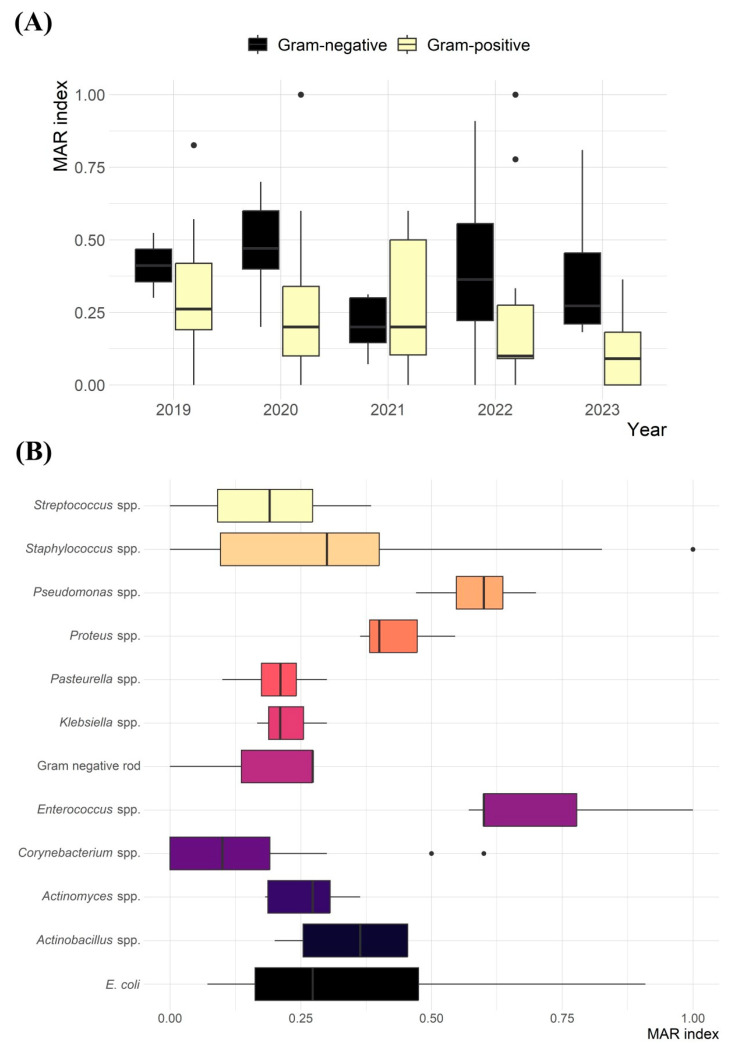
The multiple antimicrobial resistance (MAR) index calculated for bacterial isolates from 234 guinea pig clinical samples in Hong Kong. (**A**) MAR index by sampling year and Gram stain. (**B**) MAR index by bacterial species.

The average MAR index was 0.31, ranging from 0.05 to 1.00 (Figure 5). A significant difference was observed in the average MAR index between Gram-positive and Gram-negative bacterial isolates (*p* < 0.001; Figure 5A). However, no significant difference in the MAR index between isolates from 2019 and 2021 (*p* = 0.216). Significant variations (*p* = 0.001) in the MAR index were also noted among bacterial species, such as *Streptococcus* spp. versus *Pseudomonas* spp., *Streptococcus* spp. versus *Enterococcus* spp., *Staphylococcus* spp. versus *Enterococcus* spp., *Staphylococcus* spp. versus *Corynebacterium* spp., and *Pseudomonas* spp. versus *Corynebacterium* spp. (Figure 5B). Significant differences in the average MAR index were also identified between males and females (*p* = 0.021) and across sampling sites (dental versus respiratory (*p* = 0.002) and dental versus urinary (*p* = 0.003)). However, no significant difference was found in the average MAR index among different guinea pig age groups (Figure S2).

## 4. Discussion

This study analyzed data from 234 clinically ill guinea pig cases admitted to 22 veterinary clinics in Hong Kong, based on clinical records submitted to VDL, and found that the most commonly submitted samples were integument and urinary samples. The results of this study showed a high frequency of bacterial infections in clinically ill pet guinea pigs, with 57.3% of clinical samples exhibiting bacterial growth. This is notably higher than the 39.4% reported by pet guinea pig owners in the UK [7]. However, similar infection rates have been observed in other companion mammal species, such as rabbits in Hong Kong (55.8%) [4], and even higher rates have been reported in Spain (83.4%) [15].

Signalment factors such as sex and age were evaluated to assess their association with bacterial isolation rates in clinically ill guinea pigs. Although the bacterial isolation rate was higher in males compared to females, this difference was not statistically significant (*p* = 0.16), aligning with previous studies that suggest sex may not be a primary determinant of infection susceptibility in small mammals [16]. However, the higher bacterial isolation rate in males in this study could be attributed to hierarchical behavior and a tendency to engage in aggressive encounters, leading to wounds and subsequent infections [17]. In contrast, age was a significant factor, with older guinea pigs showing a markedly higher isolation rate than younger ones (*p* = 0.009). This finding is consistent with reports in other species, where age-related immune senescence or cumulative pathogen exposure may increase infection risk [17]. These results emphasize the importance of age as a critical factor in the epidemiology of bacterial infections in guinea pigs and highlight the need for targeted preventive care in older animals.

Bacterial isolation rate from integument samples was the most common (43.6%), which is consistent with previous studies that identified skin diseases as frequently diagnosed disorders in guinea pigs [18,19]. Furthermore, the bacterial isolation rate from urinary tract samples was the second most prevalent, which may be attributed to species-specific physiological adaptations in guinea pigs, such as their ability to endure water deprivation and reduced urine output, which can predispose them to urinary tract infections (UTIs) [20]. These adaptations, while beneficial in arid environments, may compromise urinary tract health by concentrating urine and reducing the frequency of voiding, thereby creating a favorable environment for bacterial colonization [21]. Similar findings have been reported in other small mammals with comparable physiological traits, where reduced urine flow and concentrated urine have been linked to increased UTI susceptibility [22]. It was also noted that most of the urinary tract bacterial isolates were recovered from females and older age guinea pigs, which aligns with findings in other species. This trend is likely attributed to anatomical differences, such as the closer proximity of the urethral opening to the anus in females, as well as age-related changes in the urinary tract and declining immune function [23].

In this study, Gram-positive bacteria (66.7%), including *Streptococcus*, *Staphylococcus*, and *Corynebacterium* spp., were the most frequently identified in samples from pet guinea pigs in Hong Kong. This finding is consistent with previous studies that reported high prevalence of Gram-positive bacteria, particularly *Streptococcus* and *Staphylococcus* spp., in small mammals [8]. In addition, *S. equi subsp zooepidemicus*, a known zoonotic pathogen, was the most frequently identified Gram-positive bacterium in this study. While *S. equi subsp zooepidemicus* is considered a normal commensal of the upper respiratory tract and oral cavity in guinea pigs, it can act as an opportunistic pathogen and has been implicated in conditions such as cervical lymphadenitis. Its presence highlights the potential public health implications of these infections, as previously reported in studies of companion animals [6]. Other bacterial species have been isolated from guinea pig clinical samples, including *S. aureus*, a common causative agent of pododermatitis, the primary integumentary disease in guinea pigs [8,18]; *P. aeruginosa*, primarily associated with respiratory infections [24]; and *C. renale*, which has been reported as the sole bacterium present in urinary calculi in guinea pigs [23].

The unique hindgut fermentation anatomy of guinea pigs and the risk of antibiotic-induced dysbiosis necessitate careful antimicrobial selection when managing bacterial infections in pet guinea pigs [25]. Our study found that 85.9% of isolates from pet guinea pig clinical samples in Hong Kong were resistant to at least one antimicrobial agent, which is consistent with findings from other studies [1,4,6]. However, susceptibility patterns varied among different bacterial species. These findings emphasize the importance of conducting AST and culture prior to antibiotic use to mitigate the development of AMR [2], and to consider the potential side effects and contraindications of these antibiotics in guinea pigs [26].

Previous literature has suggested that trimethoprim/sulfonamide, fluoroquinolones, tetracyclines, chloramphenicol, and aminoglycosides have a lesser impact on the enteric microbial flora in guinea pigs [8,27]. As a result, these antimicrobials are commonly prescribed for treating sick guinea pigs. However, this study revealed high resistance rates to penicillin, gentamicin, doxycycline, and azithromycin. It should be noted that despite the widespread use of fluoroquinolones in exotic animal practice, they are commonly considered second-tier antibiotics in small animal practice due to concerns over developing AMR [28]. The antimicrobial resistance patterns observed in this study may reflect the antibiotics commonly available and approved for veterinary use in Hong Kong. While specific data on antibiotic usage in guinea pigs are lacking, many antimicrobials used in small mammals are extrapolated from those approved for use in dogs and cats. Agents such as enrofloxacin, trimethoprim–sulfamethoxazole, and doxycycline, frequently used in exotic animal practice, were among those with notable resistance.

Our findings show that *P. aeruginosa* isolates showed higher rates of MDR isolates than other bacterial species. These results align with a study by Muñoz-Ibarra, Molina-López, Durán, Garcias, Martín, and Darwich [1], which reported high resistance levels in *P. aeruginosa* isolates from birds and mammals. Together, these findings emphasize the growing concern of AMR among bacterial isolates from exotic pets. Numerous studies have also reported high levels of AMR in *E. coli* across multiple species [4,13,29], which could be attributed to its genetic plasticity and adaptability to dynamic environmental pressures, facilitating the acquisition and accumulation of diverse antimicrobial resistance determinants and leading to a high prevalence of MDR [30]. A high frequency of MDR isolates has been reported in this study, which is consistent with the finding of a recent study in pet rabbits in Hong Kong [4]. The detection of MDR in isolates such as *Pseudomonas* spp. and *E. coli* is particularly concerning, given their potential to cause severe infections and their known capacity for acquiring and disseminating resistance genes.

The high frequency of MDR bacteria identified in pet guinea pigs in this study raises significant public health concerns. Given the close contact between companion animals and humans, there is a potential risk for zoonotic transmission of resistant bacteria or resistance genes, especially through direct handling or environmental exposure [31]. These findings highlight the importance of prudent antibiotic use in veterinary practice and reinforce the need for enhanced One Health surveillance strategies to monitor and mitigate the spread of antimicrobial resistance.

Given the unique anatomical and physiological characteristics of guinea pigs, it is crucial for veterinarians to carefully consider the choice of antimicrobials when treating bacterial infections in this species. However, the absence of complete medical histories and final diagnoses for each case, along with this study’s focus solely on aerobic bacterial infections, limits the ability to draw definitive conclusions about the overall prevalence of bacterial infections in clinically ill pet guinea pigs in Hong Kong. While there is currently no established consensus on first-line antibiotics for pet guinea pigs, the findings of this study, in combination with recommendations from previous literature, can provide guidance for selecting appropriate antibiotics based on the site of infection and bacterial morphology observed under in-house cytology.

## 5. Conclusions

This study provides crucial epidemiological insights into bacterial isolates and their antimicrobial susceptibility in clinically ill guinea pigs in Hong Kong. We found that *Streptococcus* spp., *Staphylococcus* spp., and *Corynebacterium* spp. were the most frequently isolated bacterial species from clinically ill pet guinea pigs in Hong Kong between 2019 and 2023, showing high susceptibility to ceftazidime, chloramphenicol, ciprofloxacin, and marbofloxacin. The findings aid veterinary practitioners in making informed decisions about empirical antibiotic treatment while awaiting culture and sensitivity results, supporting antimicrobial stewardship. However, the high prevalence of resistant bacteria highlights the growing issue of AMR in exotic pets and underscores the need for further research to establish evidence-based guidelines for antimicrobial use. Continued surveillance and the development of stewardship programs are essential to preserve antimicrobial efficacy and ensure the health and welfare of pet guinea pigs.

## Figures and Tables

**Table 1:** Characteristics of the pet guinea pigs population stratified by years.

Characteristics		2019 N (%)	2020 N (%)	2021 N (%)	2022 N (%)	2023 N (%)	Total N (%)
Sample		21 (9.0)	54 (23.1)	44 (18.8)	79 (33.8)	36 (15.4)	234 (100)
Sampling Site							
	Dental	1 (16.7)	1 (16.7)	--	2 (33.3)	2 (33.3)	6 (2.6)
	Gastrointestinal	2 (28.5)	--	1 (14.3)	3 (42.9)	1 (14.3)	7 (3.0)
	Integument	8 (7.8)	29 (28.4)	17 (16.7)	37 (36.3)	11 (10.8)	102 (43.6)
	Lymphatics	2 (50.0)	2 (50.0)	--	--	--	4 (1.7)
	Ocular	1 (11.1)	2 (22.2)	2 (22.2)	3 (33.4)	1 (11.1)	9 (3.8)
	Reproductive	2 (25.0)	2 (25.0)	2 (25.0)	1 (12.5)	1 (12.5)	8 (3.4)
	Respiratory	1 (5.3)	6 (31.6)	1 (5.3)	7 (36.8)	4 (21.1)	19 (8.1)
	Urinary	4 (5.1)	12 (15.2)	21 (26.6)	26 (32.9)	16 (20.2)	79 (33.8)
Sample Type							
	Swab	16 (10.3)	44 (28.2)	26 (16.7)	49 (31.4)	21 (13.5)	156 (66.7)
	Fluid	2 (8.7)	4 (17.4)	7 (30.4)	8 (34.8)	2 (8.7)	23 (9.8)
	Cystocentesis	2 (11.1)	2 (11.1)	6 (33.3)	5 (27.8)	3 (16.7)	18 (7.7)
	Voided	--	3 (10.3)	2 (6.9)	14 (48.3)	10 (34.5)	29 (12.4)
	Other (feces, fresh tissue, flush, etc.)	1 (12.5)	1 (12.5)	3 (37.5)	3 (37.5)	--	8 (3.4)
Sex							
	Male	10 (8.7)	24 (20.9)	23 (20.0)	40 (34.8)	18 (15.7)	115 (49.1)
	Female	2 (3.5)	14 (24.1)	11 (19.0)	19 (32.7)	12 (20.7)	58 (24.8)
	Male (desex)	8 (22.2)	11 (30.6)	4 (11.1)	9 (25.0)	4 (11.1)	36 (15.4)
	Female (desex)	1 (4.4)	4 (17.4)	5 (21.7)	11 (47.8)	2 (8.7)	23 (9.8)
	Unknown	--	1 (50.0)	1 (50.0)	--	--	2 (0.9)
Age							
	<1 year	5 (15.6)	5 (15.6)	11 (34.4)	4 (12.5)	7 (21.9)	32 (13.7)
	1–3 years	11 (10.0)	32 (29.1)	20 (18.2)	42 (38.2)	5 (4.5)	110 (47.0)
	>3 years	5 (5.5)	17 (18.9)	13 (14.4)	32 (35.6)	23 (25.6)	90 (38.5)
	Unknown	--	--	--	1 (50.0)	1 (50.0)	2 (0.9)
Previously Reported Antimicrobial Use							
	Yes	3 (27.3)	2 (18.2)	1 (9.1)	4 (36.4)	1 (9.1)	11 (4.7)
	No	18 (8.1)	52 (23.3)	43 (19.3)	75 (33.6)	35 (15.7)	223 (95.3)
Positive Bacterial Culture							
	Yes	12 (9.0)	35 (26.1)	23 (17.2)	38 (28.4)	26 (19.4)	134 (57.3)
	No	9 (9.0)	19 (19.0)	21 (21.0)	41 (41.0)	10 (10.0)	100 (42.7)

**Table 2:** Proportions of antimicrobial resistance in common bacterial isolates recovered from 234 guinea pig clinical samples in Hong Kong.

Bacterial Isolates	No. of Resistances						MDR n (%) ^1^	Average MAR Index (Range) ^2^
	Total N	0 n (%)	1 n (%)	2 n (%)	3 n (%)	>3 n (%)		
*Enterococcus* spp.	5	--	--	--	--	5 (100)	5 (100)	0.71 (0.57–1.00)
*Actinomyces* spp.	7	--	--	2 (28.6)	2 (28.6)	3 (42.8)	3 (42.8)	0.26 (0.18–0.36)
*E. coli *	8	--	--	3 (37.5)	2 (25.0)	3 (37.5)	5 (62.5)	0.37 (0.07–0.91)
*Pseudomonas* spp.	8	--	--	--	--	8 (100)	8 (100)	0.59 (0.47–0.70)
*Corynebacterium* spp.	27	9 (33.4)	5 (18.5)	7 (25.9)	3 (11.1)	3 (11.1)	3 (11.1)	0.20 (0.5–0.60)
*Staphylococcus* spp.	31	5 (16.1)	6 (19.4)	2 (6.4)	4 (12.9)	14 (45.2)	10 (32.3)	0.34 (0.09–1.00)
*Streptococcus* spp.	34	1 (2.9)	10 (29.5)	8 (23.5)	7 (20.6)	8 (23.5)	14 (41.2)	0.19 (0.09–0.38)

^1^ MDR = multidrug resistance; ^2^ MAR = multiple antibiotic resistance index.

## Data Availability

The data presented in this study are available upon request from the corresponding author.

## Supplementary Materials

The following supporting information can be downloaded at https://www.mdpi.com/article/10.3390/ani15142042/s1: Figure S1: Heatmap showing antimicrobial resistance patterns of the most prevalent bacterial species isolated from clinically ill pet guinea pigs in Hong Kong between 2019 and 2023. Figure S2: The multiple antimicrobial resistance (MAR) index was calculated for bacterial isolates from 234 guinea pig clinical samples in Hong Kong. (A) MAR index by animal sex, (B) MAR index by animal age group, and (C) MAR index by sampling site.

## Abbreviations

AMR	Antimicrobial resistance
MDR	Multidrug resistance
MAR	Multiple antibiotic resistance index
AST	Antimicrobial susceptibility testing
