# Supplementary material for: Distribution and Antimicrobial Resistance Patterns of Aerobic Bacterial Isolates from Clinically Ill Pet Guinea Pigs (Cavia porcellus) in Hong Kong
Source: Animals (Basel). 2025 Jul 11;15(14):2042. doi: 10.3390/ani15142042 (PMC12291934; doi:10.3390/ani15142042)

Figure S1. Heatmap showing antimicrobial resistance patterns of the most prevalent bacterial species isolated from clinically ill pet guinea pigs in Hong Kong between 2019 and 2023.

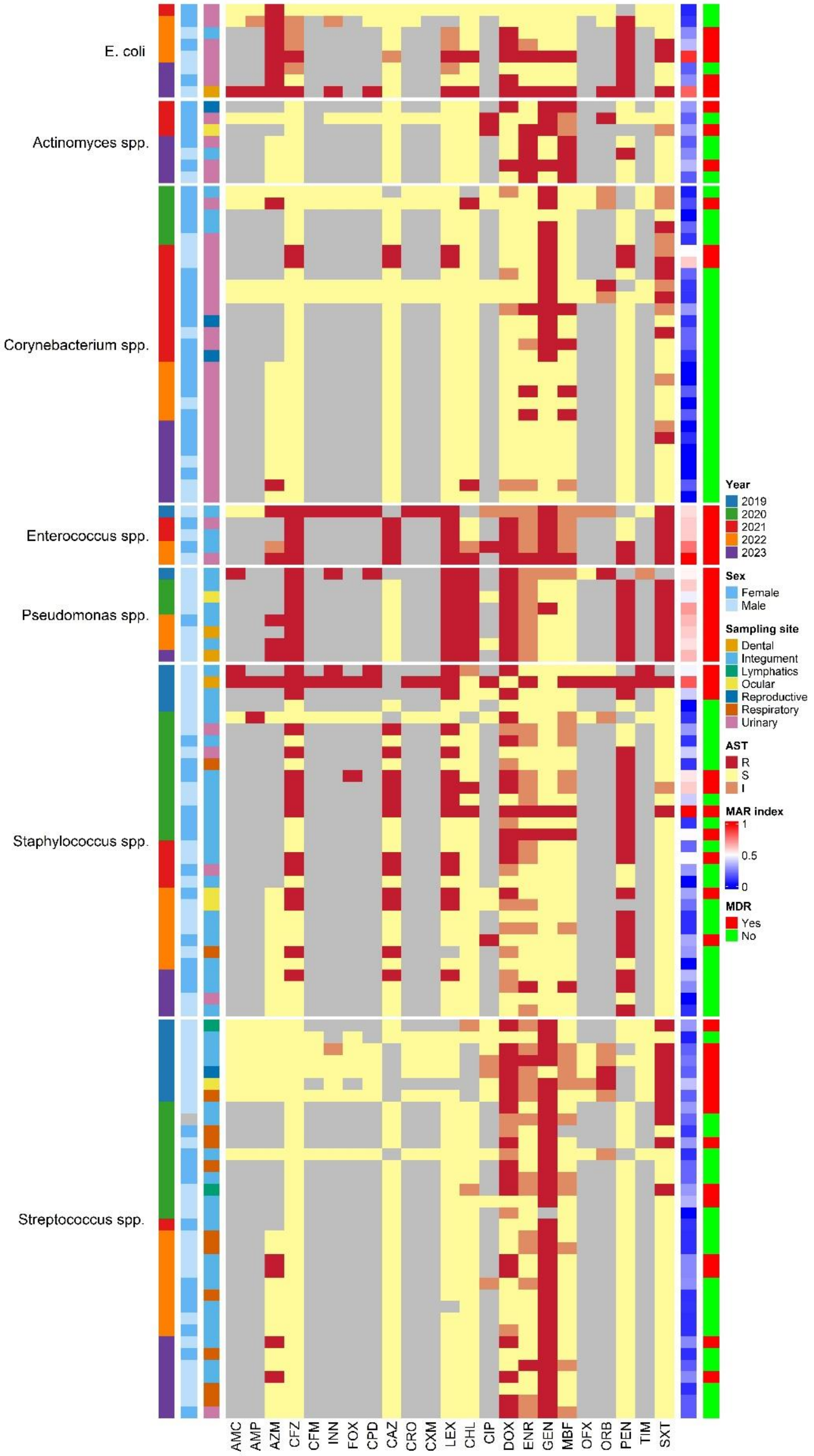

Figure S2. The multiple antimicrobial resistance (MAR) index was calculated for bacterial isolates from 234 guinea pig clinical samples in Hong Kong. (A) MAR index by animal gender, (B) MAR index by animal age group, and (C) MAR index by sampling site.

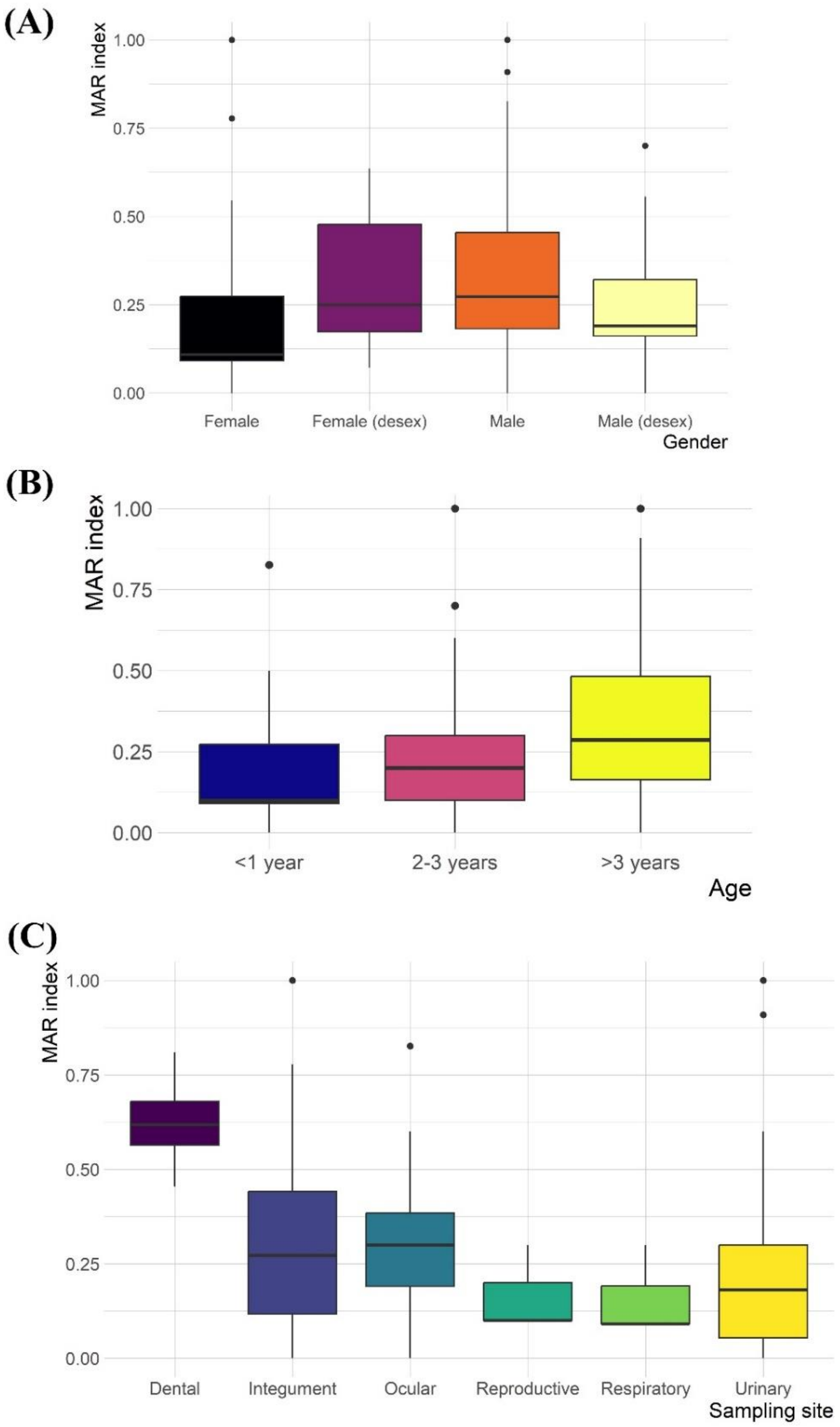

Supplement: Supplementary file 1 [file animals-15-02042-s001.zip › animals-3731936-supplementary.pdf]
